# Two Cases of Ruptured Uterus

**Published:** 1815-05

**Authors:** J. G. Coffin


*For the Medical and Physical Journal.*

### *Two Cases of Ruptured Uterus;* communicated by J. G. Coffin, M.D.

*MARCH*, 1809.—Mrs. A. aged thirty-five years, a domestic in the family of Mr. F. was taken in labour. This woman had borne several children before without any unusual occurrence, and had been as well as is common, for her state, till the night of the 12th instant, when her travail began. The membranes had given way, and the waters

 were were discharged in the night: after being for some time wet, she went at day-light from a lower to an upper apartment.

It was some hours before she could be made comfortably warm after great chilliness. I saw her at eight o'clock in the morning, when her pains were pretty frequent and severe. On examination, I could merely feel the head of the child just entering the pelvis. The contractions of the uterus, &c. continued with considerable regularity till half-after twelve, when the head had advanced to within one or two inches of the os externum.

About this time, after several pains, attended with great distress and uneasiness, the efforts of the system suddenly abated; the patient became cold, pale, and covered with sweat.

She now complained of a new kind of pain and insupportable distress about the navel, a little to the left side. These symptoms continued with unceasing severity for two hours, when she expired, before which the head had retracted some inches.

Before this accident, the bowels had been evacuated by the use of enemas. At one time I contemplated taking blood from the arm, but, as the pulse did not seem to indicate this measure, bleeding was omitted.

Immediately after the accident, every means was used to support the declining temperature and strength of the patient, and to lessen her suffering, but without any success.

The next forenoon the body was examined by Dr. J. C. Warren, who has favoured me with the following minutes of the dissection.

The appearances on the examination of the body of Mrs. —— were, as far as I can remember, the following:

The external appearance of the abdomen was irregular, from the existence of a large protuberance in the central portion of it, which was supposed to be the head of the child.

When the abdominal parietes were removed so as to lay bare the peritoneum, a dark substance was seen through the transparent membrane, on puncturing which, fluid blood was discharged from the cavity of the abdomen in great quantity. The uterus, being exposed, was found contracted to half the size it should be before delivery. Behind it lay the head of the child, which had been forced through the posterior and inferior portion of the uterus, at that part where it joins with the vagina. The placenta was also expelled into the cavity of the abdomen. The substance of the uterus, around the lacerated part, was thin, tender, and of a very dark colour. All the cellular membrane of the lower portion of the uterus was filled with blood, as well as that which which was connected with it, in every direction. The breech of the child lay in the fundus of the uterus, and constituted the tumour felt externally.

*Second Case.*—August 16, 1812.—I saw Mrs. B. this afternoon at three o'clock, and was informed that the waters of the uterus had been recently discharged. The contractions of this organ were neither considerable nor frequent; at eight her pains had gradually increased. At this time I found the os uteri pretty largely dilated, and, as I supposed, the head of the fœtus advancing, which at ten P. M. had descended to within one and a half or two inches of the os externum.

So far the progress of the labour had been slow and tedious, and the sufferings of the patient seemed to be beyond the usual degree, compared with the expulsive force of the pains. Before the hour just mentioned, I had taken a pint of blood from the arm of Mrs. B. who was a very large woman, and of a plethoric habit. Her body had been opened by injections. Soon after ten she complained of severe distress of the abdomen, accompanied with uterine hæmorrhage and sick stomach. The pains abated, and the head receded almost beyond the reach of the finger.

She vomited several times; was restless, and in great distress. In this deplorable state, apprehending the accident which had happened, I could only desire her to remain as still as possible, (for her sufferings had led her to an almost incessant change of position,) and have recourse to an anodyne.

After taking fifty drops of tincture of opium several times, she was somewhat relieved; and at four o'clock A. M. she seemed inclined to sleep. From an engagement to leave town, I was now obliged to leave the patient in the care of Dr. Gorham, who has obligingly furnished me with the following notes of the termination of the case, and of the examination of the dead body.

"August 17.—8, A. M. Was called to Mrs. B. She was sitting in an easy chair. Her respiration was somewhat hurried and laborious; the pulse was about 100, moderately full and hard; the abdomen was very tense and painful, and she complained of great thirst and flatulence. Mrs. B. had no parturient pains since Dr. C. had left her; but at 3 o'clock there had taken place a discharge of a small quantity of blood per vaginam, and another at 7 o'clock.

Examined with the hand in the vagina once two fingers in the uterus, but could feel nothing of the child.

11, A. M. Her situation is nearly the same as in the last report.

report. She feels the pressure of the child high up on the right side, and cannot lie on that side. Ordered a purgative.

Having no doubt, from the history of her case, that a rupture of the uterus had taken place, I proposed a consultation, which, from unavoidable circumstances, could not be held before 3 P. M. when, in the presence of Drs. Warren and Hayward, I passed my hand into the uterus, and moved it in various directions, meeting with no other obstructions than those arising from the sides of the organ itself, and the placenta, which was completely detached. On the posterior part of the uterus, about half way between the fundus and the neck, I perceived a longitudinal fissure, through which I passed two of my fingers into the abdomen. The same circumstances were noticed by an examination by the gentlemen who were present, and they were perfectly satisfied that a rupture of the uterus had taken place, through which the child had been forced into the abdomen. As Mrs. B. had not felt any motion in the child for twelve or fourteen hours, and as its extraction could not save its life, while, from the contracted state of the uterus, it might have proved speedily fatal to the mother, it was thought best not to attempt it. She was ordered to have an enema and anodynes.

11 P. M. Has had one copious alvine evacuation; has vomited matters of a coffee-ground appearance; bowels tense and painful; profuse sweat; pulse frequent, but firm; has been very faint.

August 13.—8 A. M. Has had no sleep; vomits continually a blackish fluid; profuse sweat, pulse very quick, thirst excessive, abdomen very tense, great dyspnœa; countenance pale and distressed; is very feeble.

8 P. P. Vomits incessantly a very dark green-coloured fluid, with flocculi of a coffee-ground appearance; pulse 115, moderately firm. Other symptoms the same as in the preceding report.

August 19.—6 A.M. Delirium; vomiting; fluttering pulse; respiration suspirious; cold sweat; in which state she continued until eleven o'clock, when she died.

*Appearances on Examining the Body at* 4 *P. M.*

*External Appearances.*—Abdomen immensely large and tense, measuring more than a yard from one groin to the other; slight discolouration about the inferior parts; on puncturing, a large quantity of very fetid air rushed out.

When the integuments, which were more than two inches thick, were divided, and the flap was laid over the pubes, the child came into view, its back presenting and lying among the intestines, which appeared to be cemented together by thick thick coagula of blood. Its head was firmly pressed under the right lobe of the liver, the left hand embracing the left foot, rested in the right iliac region; its body passed diagonally across the abdomen, the right foot being pressed against the left ileum, and the right arm forming a line with the body. The child, which was a male, was putrid, and much swelled, the cuticle separating when it was handled. When it was removed, the umbilical cord was traced to the placenta, which was found in the cavity of the abdomen, nearly at the posterior part of the uterus. The uterus itself was much contracted; its substance was very firm, and about two inches in thickness. On its posterior portion a longitudinal fissure was discovered, sufficiently large to admit three fingers; its cavity was small, but the fingers introduced per vaginam could be readily passed through the ruptured part into the abdomen. The intestines and peritoneum were highly inflamed, and there was a considerable quantity of very fetid reddish-coloured liquid in the abdomen.

J. Gorham."

*Remarks.*—Notwithstanding the diversity of opinion among accoucheurs respecting the Cæsarian operation, the following propositions seem to be sufficiently established, namely:

1st. That the operation in itself is not essentially mortal.

2d. That there are certain cases in midwifery wherein the life of the mother, of the child, or of both, depends on the removal of the contents of the gravid uterus; and

3d. That in some of these cases, this operation is the only means by which the patient can be delivered.

4th. There have been, and may again be, cases in which this operation has saved the life of the mother, of the child, or of both.

If these propositions be admitted to be true, it follows that the operation is occasionally not only proper, but also of indispensable importance.

It has been objected to the Cæsarian section, (and gastrotomy, for I include both operations, if they are to be considered as two,) that it is a hazardous operation, that it may be badly or unnecessarily performed, &c. All this is doubtless true, but these objections exist in equal force, if any, against all the higher, more difficult, and important operations in surgery. If a limb were never to be amputated, nor a stone nor a tumour to be removed, till a fortunate issue could be *certainly* foreseen, the surgeon would become a much less valuable member of society than he now is.

The truth of the second proposition, it is presumed, will be granted by all.

The third proposition is true in those instances of deformed pelvis, diseased vaginæ, and ruptured uteri, in which, from the circumstances of any of these cases, delivery through the natural passage is impossible. The last proposition is confirmed by the authentic record of many cases in which this operation has been practised with success. No reasonable objection against this resource of art can be raised from the fact that it sometimes fails, because we know it has succeeded and may again succeed in similar cases; and these successful instances may fairly be supposed to be such, as admitted of no other possible chance of saving life.

From these views of the subject, it is extremely difficult to perceive any rational foundation for the unqualified rejection of the practice in question, by Messrs. Sacombe and Simmons, or any one else. So far from agreeing with these gentlemen, I should say that this operation is not duly estimated, particularly in this country, where I cannot learn that it has ever been performed. The hour of sudden danger, painful apprehension, and alarm, is not the proper season for discussing and deciding on questions of serious import and great responsibility.

Hence the necessity that practitioners of midwifery should deliberately make up their judgment as nearly as may be, before they are called on *to act*, in what cases and under what circumstances it is requisite to have recourse to this expedient, and to be prepared to discharge their duty accordingly.

Every student of surgery should, as far as possible, familiarize his mind and his hand to the operation, under the impression that he may be required, in the course of his professional duties, not only to determine on the propriety or impropriety of this measure, where life is at stake, but also to do the operation.

How many lives have been lost from the neglect of this means of relief, we can never know; but from the limited extent to which it has been carried, and its comparative success when tried, there is no doubt in my mind that this loss has been extensive.

Nor can I hesitate to believe, that the obstetric art would be *improved*, if in all civilized communities, there should be, for the time to come, in every town or neighbourhood, one person duly qualified to decide in what cases it might be proper to adopt this expedient, and to perform the operation. It is not to be supposed that the practitioner, who has never been convinced that this operation may sometimes be necessary, who has never performed it on the dead subject, nor seen it done on the living, can be disposed and prepared to try try it, if a case should occur in which it might be unequivocally indicated.

Nor can we imagine that an individual acquainted with the operation, with a case before him in his opinion requiring it, who should recollect that it had never been done in his vicinity or country, and that all the medical men about him were opposed to it, under these discouraging circumstances should possess sufficient fortitude and regard to principle, voluntarily to encounter all the responsibility and unpleasant consequences which a discharge of his duty would be likely to involve.

Mrs. B., whose misfortune has occasioned these remarks, lived two days and a half after the rupture of the uterus took place; and the fœtus and its appendages had all escaped from this organ into the abdomen.

Supposing the fœtus, &c. to have been expelled from the uterus at the time of its division, or immediately after, as is probable, and that they had all been reasonably removed from the abdomen, by gastrotomy, is it not more probable that the patient would have survived the accident and operation, than that she should have lived sixty hours in the condition she did? It is possible, too, that the life of the child might have been preserved by a timely resort to the operation.—*New-England Journal.*

